# Design of a Multi-Epitopes Based Chimeric Vaccine against *Enterobacter cloacae* Using Pan-Genome and Reverse Vaccinology Approaches

**DOI:** 10.3390/vaccines10060886

**Published:** 2022-06-01

**Authors:** Wafa Abdullah I. Al-Megrin, Alaa Karkashan, Abdullah M. Alnuqaydan, Faris F. Aba Alkhayl, Faris Alrumaihi, Ahmad Almatroudi, Khaled S. Allemailem

**Affiliations:** 1 Department of Biology, College of Science, Princess Nourah bint Abdulrahman University, P.O. Box 84428, Riyadh 11671, Saudi Arabia; 2 Department of Biology, College of Sciences, University of Jeddah, Jeddah 21959, Saudi Arabia; askarkashan@uj.edu.sa; 3 Department of Medical Biotechnology, College of Applied Medical Sciences, Qassim University, Buraydah 51452, Saudi Arabia; ami.alnuqaydan@qu.edu.sa; 4 Department of Medical Laboratories, College of Applied Medical Sciences, Qassim University, Buraydah 51452, Saudi Arabia; ffabaalkhiel@qu.edu.sa (F.F.A.A.); f_alrumaihi@qu.edu.sa (F.A.); aamtrody@qu.edu.sa (A.A.); 5 Department of Pharmaceutical Chemistry and Pharmacognosy, College of Dentistry and Pharmacy, Buraydah Colleges, Buraydah 51418, Saudi Arabia

**Keywords:** *Enterobacter cloaca*, pangenome subtractive proteomics, immunoinformatic, molecular docking, molecular dynamic simulations, binding free energies

## Abstract

*Enterobacter cloacae* (EC) is a significant emerging pathogen that is occasionally associated with lung infection, surgical site infection, urinary infection, sepsis, and outbreaks in neonatal intensive care units. In light of the fact that there is currently no approved vaccine or therapeutic option for the treatment of EC, the current study was developed to concentrate on applications based on modern computational approaches to design a multi-epitope-based *E. cloacae* peptide vaccine (MEBEPV) expressing the antigenic determinants prioritized from the EC genome. Integrated computational analyses identified two potential protein targets (phosphoporin protein-PhoE and putative outer-membrane porin protein) for further exploration on the basis of pangenome subtractive proteomics and immunoinformatic in-depth examination of the core proteomes. Then, a multi-epitope peptide vaccine was designed, which comprised shortlisted epitopes that were capable of eliciting both innate and adaptive immunity, as well as the cholera toxin’s B-subunit, which was used as an adjuvant in the vaccine formulation. To ensure maximum expression, the vaccine’s 3D structure was developed and the loop was refined, improving the stability by disulfide engineering, and the physicochemical characteristics of the recombinant vaccine sequence were found to be ideal for both in vitro and in vivo experimentation. Blind docking was then used for the prediction of the MEBEPV predominant blinding mode with MHCI, MHCII, and TLR3 innate immune receptors, with lowest global energy of −18.64 kJ/mol, −48.25 kJ/mol, and −5.20 kJ/mol for MHC-I, MHC-II, and TLR-4, respectively, with docked complexes considered for simulation. In MD and MMGBSA investigations, the docked models of MEBEPV-TLR3, MEBEPV-MHCI, and MEBEPV-MHCII were found to be stable during the course of the simulation. MM-GBSA analysis calculated −122.17 total net binding free energies for the TLR3-vaccine complex, −125.4 for the MHC I-vaccine complex, and −187.94 for the MHC II-vaccine complex. Next, MM-PBSA analysis calculated −115.63 binding free energy for the TLR3-vaccine complex, −118.19 for the MHC I-vaccine complex, and −184.61 for the MHC II-vaccine complex. When the vaccine was tested in silico, researchers discovered that it was capable of inducing both types of immune responses (cell mediated and humoral) at the same time. Even though the suggested MEBEPV has the potential to be a powerful contender against *E. cloacae*-associated illnesses, further testing in the laboratory will be required before it can be declared safe and immunogenic.

## 1. Introduction

Antibiotic resistance (AR) is now widely acknowledged as a major danger to global mortality and mobility, resulting in enormous economic losses. Antibiotic misuse in medicines, agriculture, humans, animals, and the overall environment is the primary cause of AR [1,2]. Antibiotic resistance is an inescapable consequence of the evolutionary assumption that bacteria will acquire resistance to antibiotics as long as they are used to kill them [3,4]. New therapy methods to target AR bacteria are required to solve this alarming global issue. Among the numerous strategic approaches towards the research of antibacterial resistance, triggering human immune responses utilizing immunological therapies is an intriguing option for solving the AR issue [5,6]. Bacterial infections overwhelm the body’s natural defense mechanisms fast, limiting the treatment choices for acute infections. To treat bacterial infections, immunotherapeutic (antibodies specific to pathogens) and immunological therapies (those that regulate the innate immune response, such as novel vaccination programs) may be utilized [7,8]. Pathogen-specific immunizations might be produced using therapeutic monoclonal/polyclonal antibodies to protect susceptible populations or treat AR illnesses. There are presently no licensed antimicrobials or vaccines to combat nosocomial infections, but their introduction might drastically reduce the disease burden in hospitals [9].

Vaccines were developed in the pre-genomic period utilizing Pasteur’s vaccinology criteria, which Salk and Sabin later used to develop a safe and effective vaccine for polio [10,11,12]. Vaccines for mumps, measles, and rubella, were also developed using Pasteur’s vaccinology criteria [13]. In contrast, traditional Pasteur vaccinology failed to prevent infections that could not be cultivated in vitro because of a lack of proper culturing conditions or safety issues [14]. Furthermore, culture-based vaccines are severely limited in their application due to the large spectrum of antigenic determinants and molecular mimicry [15]. These deficiencies of conventional Pasteur vaccinology have been emphasized in the production of vaccines for commercially imperative *Neisseria meningitides* and *Mycobacterium leprae*. Because of these discrepancies, scientists are concentrating their efforts on subunit vaccine candidates that are hypothesis-driven, target the cellular components, and are generally recognized as virulence factors [16]. This is shown by meningococcal outer membrane vesicle (OMV) epidemic-specific vaccinations having porin, protein, por A, and acellular pertussis vaccines containing fimbriae and the pertussis toxin [17,18]. Such vaccine designs, on the other hand, are expensive in terms of money, time, and labour, since discovering targets that match the criteria for vaccine candidates in a big pool of bacterial pathogens is extremely improbable [19,20]. In view of these impediments, new optional and high-throughput processes were considered as ways to accelerate the development process of vaccine. This is particularly crucial when it comes to limiting the development of bacterial infections that spread swiftly [21]. At the present genomic age, the revolutions in the field of immunization using pathogen whole genome sequences and shotgun sequencing, along with bioinformatics technologies, are being employed to find likely surface-associated antigens for vaccine manufacturing [22,23]. Reverse vaccinology (RV) is a novel method of identifying surface-exposed proteins as possible vaccine targets by analyzing genetic data rather than culturing the organism [24,25]. The 4CMenB vaccine, a multicomponent meningococcal serogroup B vaccination, was developed with RV in mind. Due to the limitations in the Pasteur vaccinology approach, reverse vaccinology is a better way to prioritize good vaccine candidates in the complete proteome of bacterial pathogens [26].

The proposed work is an attempt to examine *Enterobacter cloacae* using extensive computational tools such as pan genome-based subtractive proteomics and RV [27], with the main objective of emphasizing potential vaccine targets and revealing the potential antigenic epitopes which could be used in a chimeric vaccine design against the pathogen in question, in particular, and other Enterobacteriaceae members, in general. *E. cloacae* is a Gram-negative bacterium related to opportunistic bacterial illness and has been associated with nosocomial outbreaks in hospitals over the last three decades. This pathogen may be found in both aquatic and terrestrial habitats, and it is found in the commensally micro flora of humans and animals [28]. This bacterial pathogen causes skin tissue infection, bacteremia, septic arthritis, endocarditis, intra-abdominal and respiratory/urinary tract infections, neonatal infection, osteomyelitis, etc. [29]. *E. cloacae* may potentially infect medical equipment in hospitals, resulting in epidemics. It has inherent resistance to antibiotics such as ampicillin, cephalosporins, amoxicillin, and cefoxitin, which is related to its constitutive AmpC-lactamase expression [30]. The emergence of carbapenem-resistant *E. cloacae* infections has piqued researchers’ interest in this bacterium. The lack of a vaccine for this disease exacerbates the problem of resistance, owing to ineffective preventative measures, along with the lack of a treatment, which might lead to an exponential rise in death and morbidity [31].

To investigate potential targets for designing a universally accepted chimeric vaccine, an integrative analysis covering pangenome analysis, reverse vaccinology, and immunoinformatic and structural bioinformatics was done. This was especially important owing to the lack of research on an emerging pathogen of the public health concern in the literature [32]. To increase the production of antibodies at highest level and the induction of long-lasting immune reactions, the immunoinformatic predicted epitopes were linked together to construct multi-epitope peptides [33,34]. The multi-epitope vaccine was also subjected to a three-dimensional structure prediction, and to assess the vaccine’s efficacy, we used a blind docking analysis. The complexes were also simulated to better comprehend the complex structure dynamics [35,36]. Finally, the binding free energies of the dock complex were computed to confirm the intermolecular affinity [37]. Overall, this study will update the understanding of an emerging bacterial species’ global proteome repertoire and encourage the development of innovative vaccines to combat these infections.

## 2. Material and Methods

**Figure 1:**
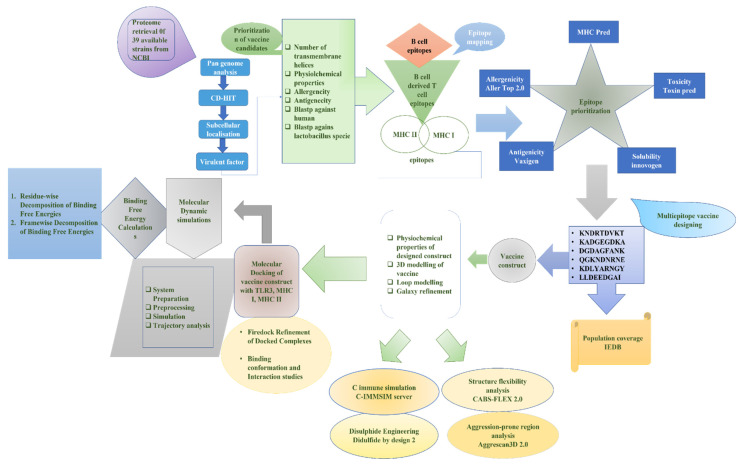
A complete methodology flow of the computational approaches designed for and applied to the protein-based MEBEPV.

The designed methodology, based on in silico for MEBEPV targeting *E. cloacae*, is presented in Figure 1, which shows the complete proteome retrieval phase, followed by the bacterial pan-genome analysis phase, redundancy analysis step, localization phase, epitopes selection designing, and processing of the multi-epitopes vaccine, molecular docking, molecular dynamic simulation, and binding free energy calculation phase.

### 2.1. A Prescreening Process for Proteome Extraction and Sub-Cellular Localization

In the beginning, the proteomic data of all of the 39 sequenced strains of *E. cloacae* [38] were retrieved from NCBI (National Center of Biotechnology Information database) [39]. The accession number of each strain of the bacteria can be found at the given link https://www.ncbi.nlm.nih.gov/genome/1219?genome_assembly_id=212284 (accessed on 1 November 2021). Bacterial pan-genome analysis was conducted on retrieved proteome. A BPGA standalone tool identifies proteins that are common in all pathogens, as well as relatively conserved in order to prioritize prospective vaccine candidates for development. CD-HIT analysis was then performed on the core proteome in order to shortlist the protein sequences considered as non-redundant and having the sequence identity with a threshold value of 90 percent based on the sequence similarity cut-off values. The paralog proteins were then subjected to surface localization analysis, which was done using the online webserver PSORTb [40,41].

### 2.2. Predicting Pathogenic Proteins—A Vaccine Candidate Prioritizing Stage in the Early Development Stage

A large role in pathogenesis is played by virulent proteins, which are thus excellent targets for vaccine candidates. The virulent factor database (VFDB) computes the pathogen proteins within the core proteome of chosen pathogens, and proteins are categorized as virulent when having >30 percent identity and a greater than 100 bit score [42]. The characterization of physiochemical properties and the selection of pathogen exoproteomes and secretomes were carried out using a ProtParam tool in order to make the experimental tests of the vaccine more straightforward. In selected proteins, the number of transmembrane helices was determined using HMMTOP 2.0, (http://www.enzim.hu/hmmtop/html/document.html accessed on 13 November 2021) and proteins that contained ≥ 1 TM were then selected for further investigation. Proteins with molecular weight (MW) of less than 110 kDa are were likely to be considered as potential vaccination targets. The antigenic tendency of the chosen proteins were verified using the VaxiJen server, and thus the proteins found to be non-antigenic were eliminated. To conclude, the proteins that had been screened and showed highly antigenic tendencies against T-cell receptors, as well as antibodies, were evaluated as promising targets. To estimate the adhesive properties of all of the chosen proteins, the SPAAN server with the default parameters was used. This stage was critical in determining the viability of prospective vaccine candidates being powerful mediators of pathophysiology by generating disease due to their sticky properties. Moreover, homology screening of potential candidates against the human proteome to examine non-host homologous proteins was performed using the NCBI (National Center for Biotechnology Information). The following input parameters were used: an E-value of less than one, a bit score of more than 100, and a sequence identity of greater than thirty percent. Homologous proteins were excluded from the experiment in order to prevent the host’s autoimmune response [42].

### 2.3. Mapping T Cell Epitopes Obtained from B Cells

The resulting proteins were submitted to epitopes prediction by the “immune epitopes database (IEDB)”, which included sequences predicted to be associated with B cell-derived T cell epitopes. Linear B-cell epitopes were predicted through the “Bepipred Linear Epitope Prediction 2.0 in IEDB” software package [43,44]. Following that, T-cell epitopes mapping was carried out using selection criteria with a score of greater than 0.5. With the use of a percentile score, it is possible to identify epitopes that bind with alleles, specifically “HLA II (DRB*0101)”, which is present in the major MHC- and II complexes immune receptors, which are divided into MHC classes I and II. Only those epitopes having the lowest percentile scores were filtered out and designated as having a high-affinity binding sequence (HABS). A further step was taken to refine the binding affinity of the preferred epitopes using the MHCPred 2.0 program (http://ddg-pharmfac.net/mhcpred/MHCPred/ accessed on 26 November 2021), and only those having less than 100 mM inhibitory concentrations (IC_50_) for the main allele HLA II DRB*0101 were considered to be potent binders for the selected alleles [45]. The predicted epitopes (B and T cells) were tested using the VaxiJen, AllerTOP 2.0, and VirulentPred programs (accessed on 4 December 2021). Toxin-pred was used to assess the toxicity for all non-allergenic filtered epitopes, and those non-toxic epitopes that had been acquired were then submitted to the IFN epitope server to determine if they had the ability to trigger IFN-γ [46]. Finally, the sorted epitopes were submitted to the IEDB epitope conservation analysis method to estimate their conservation across the selected MDR pathogens to develop a universal vaccine design strategy. The completion of this phase is vital in the development of a universal potential vaccine that has the potential to target multidrug-resistant strains [47].

### 2.4. Designing and Evaluating the MEBEPV Systems

**Figure 2:**
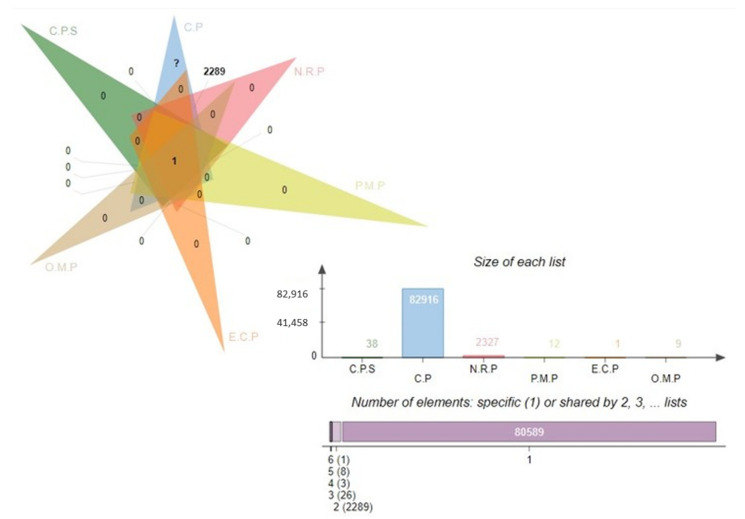
Venn analysis of the subtracted proteins in each subtractive filter. Complete proteome sequence, C.P.S; core proteome, C.P; non-redundant proteins, N.R.P; periplasmic membrane proteins, P.M.P; extra cellular proteins, E.C.P; outer-membrane proteins, O.M.P.

As a consequence of proteomic mapping, all of the resulting B-cell-derived T cell epitopes were linked with a GPGPG linker to form multi-epitope-based *E. cloacae* peptide vaccine constructs (MEBEPV). A linker EAAAK was used for binding an adjuvant (-defensin) to the vaccine’s N-terminal, which was then administered to patients. To enhance the immunogenicity of the MEBEPV, an adjuvant was added, as shown in Figure 2. To determine the physiochemical features of the MEBEPV, i.e., its molecular weight (MW), instability index (II), alphabetic index (AI), theoretical isoelectric point (PI), and GRAVY (grand average of hydropathicity), Protparam, an online server, was utilized. Tests on the allergenicity and antigenicity of MEBEPVs were conducted using the VaxiJen 2.0 and AllerTOP webservers [48,49]. When it comes to identifying protein folding, SOPMA was employed to study the MEBEPVs’ secondary structure. From the SCRATCH suite, SOLpro tool was also used to forecast the solubility of the vaccine in water. The three-dimensional (3D) structure for the MEBEPV was modeled using 3Dpro, which is part of the SCRATCH protein server. The predicted 3D structure was next proceeded for loop modeling using GlaxyLoop, and subsequently refined using GalaxyRefine, which is a step-by-step process [50]. Afterwards, the revised protein model was evaluated for the residue pairings that were suitable for disulfide engineering using the disulfide engineering method. It was decided to undertake disulfide engineering for an improved structure of the MEBEPV by disulfide design 2.0 in order to cause improvement in the overall structure’s stability [50]. Disulfide engineering is a technique for simulating disulfide links inside a protein’s structural framework. Afterwards, the “Java Codon Adaptation Tool (JCat)” was applied for the MEBEPV codon optimization approach to ensure higher expression while utilizing the *E. coli* K12 expression system as a reference, which was previously described [51]. For this aim, the MEBEPV sequence was reversibly transformed into another sequence. The overall GC content and CAI of optimized nucleotide sequences were determined by the server, and these values indicated the expression amount in the *E. coli* expression system. Ultimately, the optimized DNA sequence was being cloned into the “pET28a (+)” plasmid with the help of the Snap Gene tool [52].

### 2.5. Immuno-Profiling of Universal MEBEPVs In Silico

The C-ImmSim online webserver was used based on a position-specific matrix (PSSM) to determine inducing a host immune response against the designed construct. The following were the input parameters that were used to stimulate the immune response simulation: the volume was set to 10; the number of injections was set as 1; the number of steps were set as 100; the random seed selection was 12,345; and the HLA was set to A0101, A0101, B0702, B0702, DEB1 0101, and DRB1 0101. Aside from that, all other factors remained the same [2,53].

### 2.6. Docking and Enhancement of MEBEPVs Using Blind Docking

Ideally, the designed multi-subunit vaccine should interact well with the receptors and host immune cells in an effective manner in order to elicit the appropriate immunological response. An investigation of the binding affinity of the vaccine construct with the receptors of the human immune system was conducted via molecular docking analysis. A server named PatchDock was used to execute the blind docking of MEBEPVs with selected immune receptors, which included MHC-I, MHC-II, and TLR-3, in order to determine their interactions [54]. From the protein data bank (PBD), the three-dimensional structures of the receptors were obtained. The shape complementarity concept was used to examine the interactions between all of the residues. This blind docking approach was a key step in determining the structure of the vaccine construct, along with short listing epitopes that possibly had a high binding interaction with the selected immune receptors throughout the vaccine development process. It was then necessary to cluster the PatchDock complexes using an RMSD set to 4.0 by default, and the resulting protein–protein docked complex was submitted to a server called FireDock for refinement purposes [55]. It is possible to generate sophisticated PatchDock complexes using this server, which is a powerful platform. Following that, all of the complexes were selected with the lowest global energy and chosen for the high-throughput conformational investigations of the MEBEPV using the UCSF Chimera 1.13.1 software. The mechanism of the MEBEPV binding and the intermolecular forces between the immunological receptors (TLR3, MHCI, and MHCII) and the vaccine construct were investigated in this manner [56].

### 2.7. CABS-Flex and Aggregation-Prone Zone Investigations

The examination of the vaccine’s structural flexibility was necessary to comprehend in order to ensure adequate functionality and molecular recognition. In order to accomplish this, the CABS-Flex 2.0 web server was used to run a coarse-grained simulation of both vaccines that were being developed simultaneously. The following parameters were used in the simulation process: the number of cycles (50), the RNG seed (4257), the number of cycles between trajectories (50), the weight of the global C-alpha restraints (1.0), and the weight of global side chain restraints (1.0). The Aggrescan3D 2.0 program also helped in the identification of aggregation-prone locations in the vaccination candidates.

### 2.8. Molecular Dynamics Simulation (MDS) as a Method of Testing

All of the docked complexes that were chosen were then subjected to an MD simulation experiment in a multi-step procedure to determine their dynamics and stability. The system preparation was divided into three phases: (1) pre-processing, (2) production, and (3) post-production. “AMBER18” was applied for the execution of the 50-ns simulation of the docked complexes, and the results were published [57]. The first phase was accomplished via the use of an AMBER18 antechamber module and complicated libraries, as well as different parameters intended for each of four immune receptors, were chosen (TLR3, MHC I, and MHC II), and the vaccine build was obtained and used in the second phase. The leap module of the AMBER program was used to solvate the complicated system in the TIP3P solvation box with the input value equal to size 12 (as the size of the solvation box). During the MD stimulation test, an ff14SB force field was used to investigate intermolecular, as well as intermolecular interactions, between molecules. In order to achieve charge neutralization, Na+ ions were injected into the solution as counter ions. In the pre-processing phase, the energy was increased, being associated with the complex by running numerous rounds in the following way: the energy minimization of hydrogen atoms (500 runs) of the water box (1000 runs, energy restraint of 200 kcal/mol—A2 on the rest of system), the minimization of the atoms of the complete system (1000 runs, but with a restraint of 5 kcal/mol—A2 on C atoms), and non-heavy atoms. The complex was then exposed to a heating procedure by an NVB ensemble at a temperature of 300 K, with the temperature sustained at that degree for stabilization of the hydrogen bonds (HBs), which was performed utilizing the SHAKE algorithm and Langevin dynamics [58]. For the complex to achieve pressure equilibrium, pressure settings equal to 100 ps were applied and maintained using the NPT ensemble tool with a 2 s time scale. Such a technique allows for the establishment of a restriction on the number of C atoms with 5 kcal/mol—2 binding energy [59]. Consequently, a statisticall computational technique used to separate and sort out non-bonded forces utilizing the CPPTRAJ module and a default threshold value set for an 8.0-micron distance as the basis for the separation and filtering. This phase was critical in order to investigate the stability of the complexes in relation to various structural factors and to evaluate the stimulation trajectories on the complexes produced for the proposed MEBEPV with four TLRs. To conclude, the software used to visually analyse all of the various MD stimulation paths that had been created was Visual Molecular Dynamics (VMD) 1.9.3 [60]. With this assay, we were able to assess the interaction of the MEBEP (all TLRs) receptors as a time and function and monitor the overall epitopes that remained accessible to these immune receptors of the host, which was crucial for both detection and long-term pathogenicity [61].

### 2.9. Immune Receptors-MEBEPV Complexes Were Subjected to Binding Free Energy Measurements in Order to Determine Their Binding Affinity

In order to quantify the related binding along with solvation free energy created due to interactions of the MEBEPV with immunological receptors, MHC I, MHC-II, and TLR-3, we used the MMPBSA.py module in AMBER 16 and the MMPBSA.py module in the MMPBSA.py module [61]. To calculate the binding free energy, 100 frames were selected from the molecular dynamic simulation’s total length and processed in the usual way. In order to calculate the overall binding energy for the docked complexes, the following equation was used:

G bind = G bind, vaccum + Gsolv, TLR, − MEBEPV − (G bind, MEBEPV + Gsolv, TLR) − (G bind, MEBEPV + G bind, TLR)

## 3. Results

### 3.1. Pan Genome Analysis and Retrieval of the Core Proteome

The core proteome for *E. cloacae* consists of 82,916 proteins, which is a large number. It was determined that a subtractive proteomics framework might be utilized to examine the core proteome in order to forecast possible vaccination candidates against specific bacteria [39]. To eliminate redundant proteins, CD-HIT analysis was performed. Given the existence of redundant databases that could induce biases and make the procedure in in silico inexpensive, a redundancy analysis is an unavoidable requirement in bioinformatics approaches. In the core proteomic dataset, 2327 non-redundant proteins were identified using this redundancy filter, each of which was represented uniquely. The subcellular distribution of non-redundant proteins indicated the presence of 12 periplasmic proteins, 1 external protein (exoproteome), and 9 outer membrane proteins (secretomes), as mentioned in the Figure 2 Venn. A host’s immune system recognizes antigenic epitopes on the surface of proteins (periplasmic, outer membrane, or extracellular) and directs immune responses against these antigens. Furthermore, such proteins are important virulence promoters for pathogens since they allow for adhesion to host tissue, intracellular survival and proliferation, the invasion of host cells, and the onset of disease in susceptible individuals.

### 3.2. Physiochemical Characterization and Evaluation of E. cloacae Being Antigenic

Different physiochemical characteristics, antigenicity, and the number of transmembrane helices of the pooled pathogenic proteins were assessed to select the potential vaccine candidates for further evaluation and vaccine development [40]. The findings of this study were utilized to help choose suitable vaccine candidates for experimental testing that might be used for swift vaccine development. The molecular weight (MW) of proteins was one of the first factors to be explored. The effectiveness of prospective vaccination targets has been shown to be enhanced when proteins with a MW of 110 kDa are used. When it comes to structural and functional investigations, proteins with a smaller molecular mass are easier to identify and purify. In a nutshell, all of the pathogenic proteins were found to have a molecular weight (MW) of less than 100 kDa, and among them, 12 proteins were discovered to have TM-helices of less than one, and these proteins were considered for further investigation. Because of the anticipated difficulty during the extraction process of protein, purification, cloning, and expression, proteins having numerous TM helices were typically supposed to be less-effective candidates for the vaccine. During the process of identifying prospective vaccine candidates, it was also important to assess the protein’s stability. Aside from the extracellular protein, it was discovered that all periplasmic and outer membrane proteins were stable. For pathogenic proteins present in diverse locations, the average theoretical isoelectric point (pI) estimated was 4.6 for extracellular proteins, 4.83–9.08 for periplasmic proteins, and 4.47–7.73 for outer membrane proteins. Although the pI values are difficult to quantify, they are a valuable indicator of the likelihood of locating a protein of interest on a suitable portion of 2D gel. In the case of periplasmic proteins, the average GRAVY values were −0.26, −0.31, and −0.41, as well as in the case of extracellular proteins, respectively. The hydrophilic character of proteins is indicated by a negative GRAVY value.

Each protein was subjected to additional analysis using the VaxiJen server, which identified six, one, and six antigenic proteins from the periplasmic protein’s datasets, the extracellular proteins dataset, and the outer membrane proteins dataset, respectively. Adhesive probability was evaluated for 13 antigenic proteins, and only eight proteins were discovered: >core/6131/1/Org1 Gene1328 (adhesion score, 0.691), >core/7847/1/Org1 Gene1898 (adhesion score, 0.773), >core/2610/29/Org29 Gene4218 (adhesion score, 0.793), and >core/1134/29/Org29 Gene2696). It was discovered that the remaining five proteins did not adhere to one another, and they were therefore eliminated from further consideration. Of the eight sticky proteins tested, two were discovered to be allergens (core/2610/29/Org29 Gene4218 and core/8580/1/Org1 Gene1331) and three were found to be weakly water soluble and so filtered out (core/7847/1/Org1 Gene1898, core/313/2/Org2 Gene3708, and core/313/32/Org32 Gene4550). The discovery and subsequent elimination of human-related proteins was accomplished through the use of the BLASTp program from the NCBI data store. During the epitope mapping process, one homologous protein was identified with 32 percent similarity and eliminated, while two non-homologous target proteins (BBS36577.1 and WP 038419991.1) were discovered with no similarity to the Lactobacillus species and were therefore included in the epitope mapping process shown in Table 1.

### 3.3. Epitopes Prediction Phase

**Figure 3:**
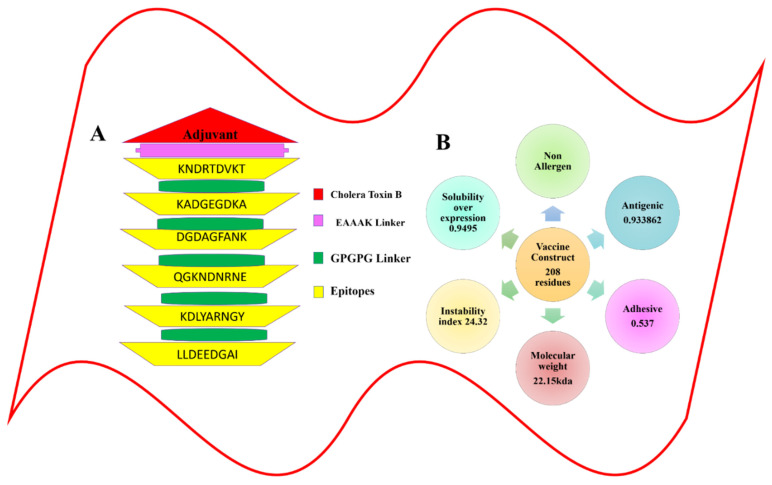
Graphical representation for the multi-epitope-based peptide vaccine construct. (**A**) Schematic diagram of the multi-epitope-based peptide vaccine construct. (**B**) The physiochemical properties calculated for the multi-epitope-based peptide vaccine construct.

In the analysis of two vaccination candidates for B and T cells epitopes prediction, 18 peptides with a threshold of >0.5 were identified, and these peptides were shortlisted as good binders. Because the immune system’s defense mechanisms are generated by the binding of B cell epitopes to specific antibodies, it is critical to predict B cell epitopes before they are discovered [62]. This discovery was made when the B cell peptides were screened for potential T cell epitopes and found to contain the MHC I and MHC II binding sites. The T cells with the CD8 antigen receptor identify the MHC I molecules, showing their presence on the nucleated cell surface, causing an immediate immune response that kills the presenting cells. In contrast, the MHC II molecules are found on the antigen-presenting cells of the immune system (APCs) and are recognized by the CD4+ T cells as antigens. A total of 50 B-cell-generated T cell epitopes were chosen from the pool of two priority proteins. Additionally, the MHCPred pipeline was performed in order to identify the epitopes with the maximum level of binding potency to the “DRB1*0101* allele”, which is widely distributed across the world population, and epitopes that bind to this allele have the potential to elicit robust immune responses [63]. It was determined that this efficacy of binding was 50% in terms of the IC50 value. Generally speaking, the lower the IC50 number, the better the quality of the forecast. A total of 23 epitopes with IC50 values of less than 100 nM were chosen. VaxiJen 2.0 predicted the antigenic properties of the selected epitopes based on their sequences. Only 19 epitopes with an antigenicity score of greater than 0.7 were considered for inclusion since they were expected to have the ability to bind to the antigen. In order to improve the precision of epitope selection, epitopes that were antigenic in nature were further screened for the presence of other physiochemical features [64]. All 13 allergenic epitope sequences were deleted in order to rule out the possibility of an allergic reaction, and 6 epitopes were determined to be non-allergenic. These were then tested for toxicity in order to avoid the potential toxicity associated with the vaccination. Finally, six antigenic, non-allergen, non-toxin, and soluble (KNDRTDVKT KADGEGDKA, DGDAGFANK, QGKNDNRNE, KDLYARNGY, and LLDEEDGAI) epitopes were chosen for further investigation, as shown in Table 2 and schematically represented in Figure 3.

### 3.4. Globall Population Coverage of the MHC I and MHC II Molecules

**Figure 4:**
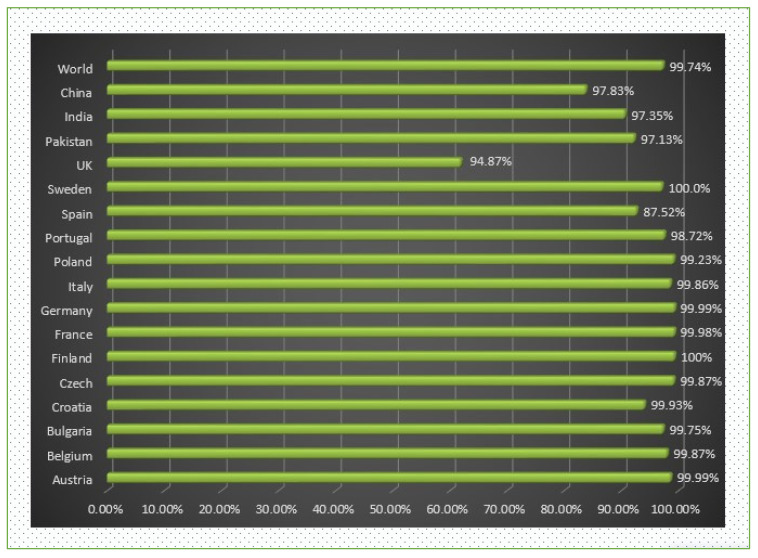
World- and different country-wise population coverage.

**Figure 5:**
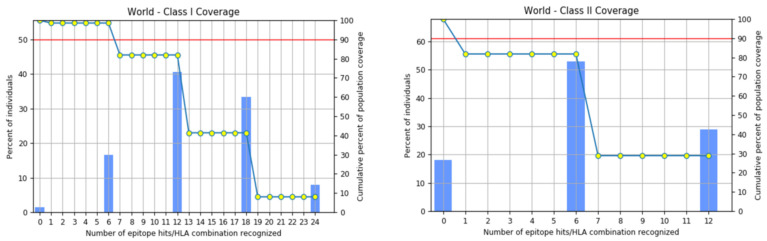
Worldwide coverage of MHC classes I and II allele HLA perceived as a set of predicted epitopes of T cells. Graphs for the worldwide class I and II populations coverage represent the relationship between the number of individuals and the number of epitope–allele combinations. The cumulative percentage for the MHC I coverage is observed as 98.98%, while for MHC II it is 94.71%.

When predicting the combined MHC-I and MHC-II population coverage for the final selected epitopes against a set of alleles covering geographic areas around the world, the IEDB analytic tool generated a worldwide population coverage estimate. The results have shown that MHC class I has the largest population coverage, with 98.98 percent, and MHC class II has the second highest population coverage, with 94.71 percent. Furthermore, according to the server, the anticipated values for the PC90 (the number of epitope–allele combinations recognized by 90% of the world population) for MHC-I and MHC-II were 15.14 and 5.5, respectively, showing the population targeted by the designed vaccine for different geographic regions of the world (shown in Figure 4 and Figure 5). We concluded that certain epitopes may be viewed as possible aspirants and should be explored for inclusion in the creation of a multi-epitope vaccine design.

### 3.5. Physiochemical Properties and the Design of the MEBEPV

The MEBEPV was created by joining together six possible epitopes using the GPGPG linker. The N-terminal of the proposed vaccine construct, which consists of a lengthy chain of 208 amino acids, has an EAAAK linker connected to it with an adjuvant (cholera toxin B). Following the design of the MEBEPV, it was subjected to further testing for physiochemical and immunogenic profiles. To begin with, the MEBEPV was compared to the human proteome in order to rule out any similarities. It was then determined whether MEBEPV was allergic and antigenic, and it was discovered that the proposed vaccine design is likely to be non-allergenic and highly antigenic, with an antigenic score of 0.933. They were confirmed to be 6.43 and −0.585, respectively, in terms of their theoretical pI and GRAVY values. If a GRAVY value is negative, it suggests that the intended construct is hydrophilic. As well as being thermally stable, its low molecular weight (22.15 kDa) will make it useful for experimental evaluation. The solubility of designed vaccine construct is 0.673

### 3.6. Structure Prediction and Refinement

**Figure 6:**
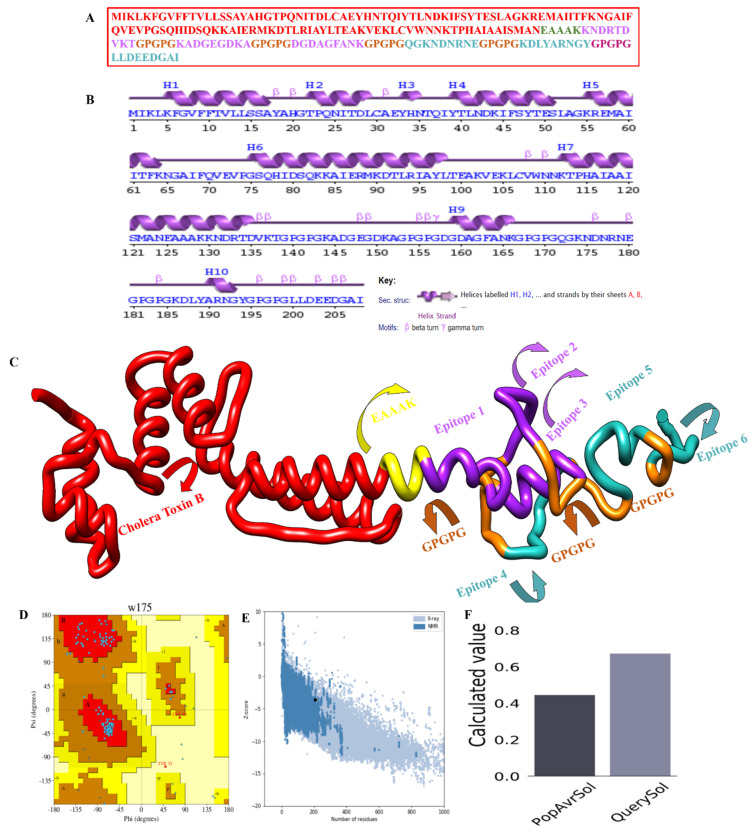
Structural classification of the multi-epitope-based peptide vaccine construct: (**A**) polyepitope vaccine construct sequence, (**B**) secondary structure for the MEBEPV, **(C)** Tertiary structure for the MEBEPV, (**D**) Ramachandran plot of the selected model, (**E**) ProSA-web results presenting the Z-score, and (**F**) solubility graph.

Whole-organism and large-protein-based vaccinations have proven to be particularly effective in lowering infectious disease-related mortality and morbidity [65,66,67]. Nevertheless, the next stage was to create a 3D structure of the designed vaccine, as shown in Figure 6C, using the “SCRATCH” webserver server’s 3Dpro, which was then loop-modeled using GlaxyLoop. A total of six (GLU50-MET89, GLN70-PRO75, CYS30-ILE38, ASP135-PRO154, GLY155-LYS174, and ASN175-TYR194) loop refinement runs were executed. Then the loop-modelled construct was uploaded to the GalaxyRefine server for refinement. Model 5 was chosen because it had the lowest MolProbity clash score, stable energy, and a maximum number of Rama-preferred residues, with no poor rotators in the refined model. According to the Ramachandran plot analysis (Figure 6D), Model 5 had poor rotators (0.6); a high Rama-favored region (97.6); overall acceptable GDT-HA (0.9435), RMSD (0.435), and MolProbity (1.604); and a lower clash score (9.8), as shown in Table 3. Figure 6E highlights the Z-score for the residues. the solubility score is represented in Figure 6F, and the highest score is observed as 0.6.

### 3.7. Analysis of the CABS-Flex and Aggregation-Prone Regions

**Figure 7:**
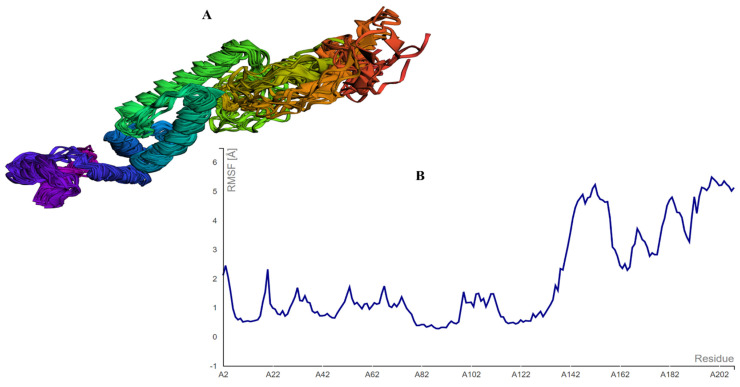
CABS-FLEX analysis showing (**A**) the model obtained by the simulation, and (**B**) the largest RSMF with a 5.489 score.

The MEBEPV was also subjected to a CABS-Flex study, which resulted in the creation of ten models after the simulation. The largest RMSF discovered was 5.489, while the least RMSF discovered was 0.2800. This means that the structure having less residual flexibility has greater stability than before. The CABs-Flex analysis is provided in Figure 7.

### 3.8. Disulfide Engineering, In Silico Cloning, and C-Immune Simulation

**Figure 8:**
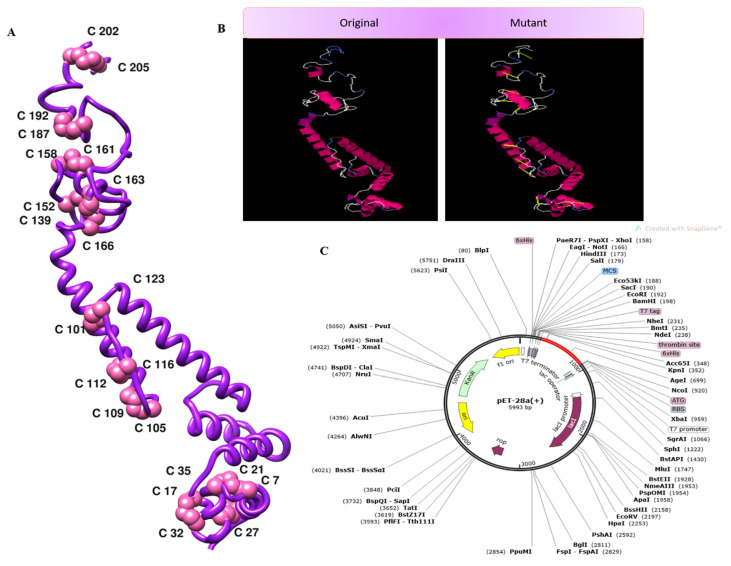
(**A**) 3D structure of the mutant vaccine. (**B**) Mutant and wild vaccine. (**C**) Translated DNA sequences cloned in the pET28a (+) plasmid.

**Figure 9:**
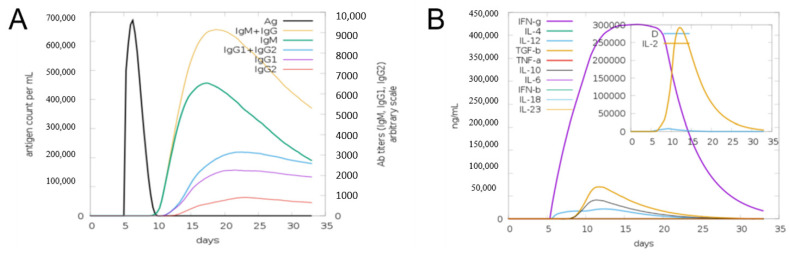
Computational immune simulation of the host immune systems obtained in response to the action of the original design of the vaccine. (**A**) The production of immunoglobulins in response to the originally designed vaccine can be seen. (**B**) Graph depicting the activation of the immune system, as the interleukin and IFN-g production can be observed.

The disulfide engineering approach of the vaccine design was conducted in order to reduce the conformational entropy, which results in increased stability of the folded orientation of the vaccine, as mentioned in Figure 8A. Disulfide bonds were found in both the inter- and intra-chains throughout the screening process. It was discovered that 14 pairs of residues were capable of being mutated. Thereweare 14 pairs of genes that were chosen to be altered, as follows: LEU4-GLY7, THR11-THR-27, ALA17-GLY-21, GLU32-ASN-35, ALA101-ALA123, LYS105-ALA-116, TRP109-LYS112-ALA152-PHE163, ASP158-ALA161, ASP187-ASN-192, and ASP202-ASP205. Figure 8B depicts the disulfide bond-containing mutant and the original structures of the vaccine construct to disulfide bonds. Additionally, the JCat tool was employed to evaluate the cloning and expression of the vaccine construct using an expression vector. The GC content was 51.7% and CAI was 0.96% in JCat, indicating that the expression system in the *E. coli* system is at a very high level. It was necessary to clone a vaccine construct gene into a pET28a (+) plasmid in order to express it in *E. coli*, and therefore the sites of restriction were added at the 5 and 3 ends of the sequence [51]. The sequence (GCTAAAGTTGAAAAACTGTGCGTTTGGAACAACAAAACCCCGCACGCTATCGCTGCTATCTCTATGGCTAACGAAGCTGCTGCTAAAAAAAACGACCGTACCGACGTTAAAACCGGTCCGGGTCCGGGTAAAGCTGACGGTGAAGGTGACAAAGCTGGTCCGGGTCCGGGTGACGGTGACGCTGGTTTCGCTAACAAAGGTCCGGGTCCGGGTCAGGGTAAAAACGACAACCGTAACGAAGGTCCGGGTCCGGGTAAAGACCTGTACGCTCGTAACGGTTACGGTCCGGGTCCGGGTCTGCTGGACGAAGAAGACGGTGCTATC) was cloned into the pET28a (+) vector (as seen in Figure 8C) using the SnapGene program, and the resulting clone was 5993 bp in length. Furthermore, the production of antibodies in the form of “IgM and IgG” was also seen in conjunction with a high level of the MEBEPV antigen processing and expressing to the human immune system for a continuous 5 days was detected. In addition to secondary immune reactions, there are also tertiary immunological responses, which result in heightened titer of “IgM IgG, IgM, IgG1+IgG2, IgG1, IgG2” and a large population of B cells, among other things. Similarly, for nearly 32 days, there was an exceptionally significant escalation in interferon gamma IFN-g (> 300,000 ng/mil), which is a marker of inflammation. All the immune responses are shown in Figure 9.

### 3.9. Binding Analysis of the Designed Vaccine to MHC-I, MHC-II, and TLR-3

**Figure 10:**
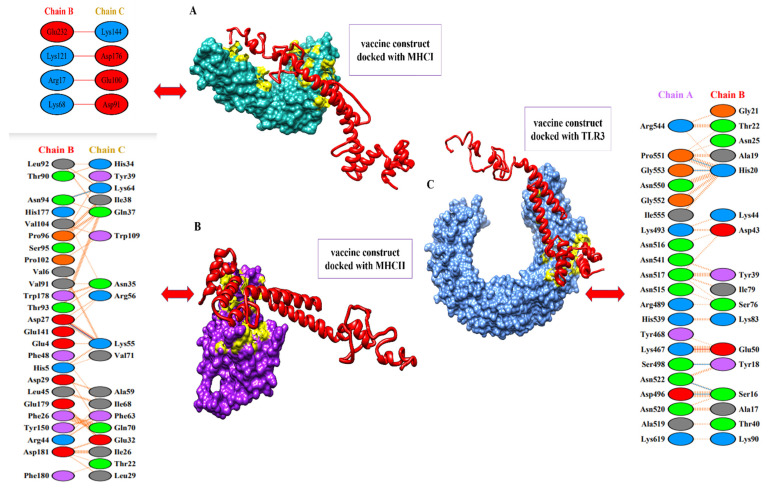
Docked complexes of the vaccine to MHC-I, MHC-II, and TLR-3 receptor molecules. (**A**) The designed vaccine construct with the MHC-I molecule, along with interactive amino acid residues. (**B**) The image represents the designed vaccine construct with the MHC-II molecule, along with interactive amino acids residues. (**C**) The image represents the designed vaccine construct with the TLR-4 molecule, along with interactive amino acids residues.

To activate the immune responses, a potent vaccine should have good binding affinity with the immune receptors of hosts, such as TLR3 and MHC molecules. With the help of a protein–peptide molecular docking approach, the best matching connections of MEBEPVs to MHC I, MHC II, and TLR3 receptor molecules were determined. A set of the top ten models based on protein surface shape and electrostatic complementarity was produced based on the PatchDock results, and these models demonstrated significant binding interactions. The molecular docking solutions were rescored and refined with the help of the Fire-Dock server. On the basis of the binding score, we chose the ultimate top model from the potent 10 models. From the docking results, the top ten models based on geometry and electrostatic complementarity of protein surface were generated, and they showed strong binding interactions. The Fire-Dock server was also used for refinement. Amongst the potent ten models, the final top model was chosen based on its binding score. Solution nine of the docked complexes (MEVC-TLR3) has shown a global energy of −5.20 kJ/mol, a repulsive van der Waals of 13.08 kJ/mol, an atomic contact energy of 17.38 kJ/mol, and a hydrogen bond energy of −7.28 kJ/mol, while solution seven of the docked complexes (MEVC-MHC I) has shown a global energy of −18.64 kJ/mol, a repulsive van der Waals of 11.75 kJ/mol, an atomic contact energy of 3.46 kJ/mol, and a hydrogen bond energy of −2.04 kJ/mol. Furthermore, solution two of the docked complexes (MEVC-MHC II) has shown a global energy of −48.25 kJ/mol, a repulsive van der Waals of 25.59 kJ/mol, an atomic contact energy of −2.59 kJ/mol, and a hydrogen bond energy of −1.70 kJ/mol. The statistics of the docking assays are shown in Table 4, and docking images are given in Figure 10.

### 3.10. MDs Simulation of TLRs-MEBEPV Complexes

The RMSD analysis of the systemic Cα atoms of the docked complexes revealed that the maximum trajectory of the MHC-1 complex (black colour in the graph) was 3.5 nm, that of the MHC-II complex (green colour in the graph) was 1.7 nm, and in the TLR3 complex (red colour in the graph), the maximum trajectory was held at 4.5 nm during the simulation run. The RMSD study found that the MHC-1 complex initially rose to 3.2 nm until 15 ns, then it dropped to around 3.5 nm after 20 ns and stabilised at about 3.5 nm at the end of the simulation. Similarly, the MHC-II complex steadily escalated to roughly 1.7 nm after 25 ns and remained stable, with minor fluctuations, until 50 ns. The TLR3 complex had a slow rise to 3.8 nm after about 20 ns, then stayed at an average of 4 nm until 35 ns, after which it rose to 4.5 nm until 40 ns, and then dropped to 3.5 nm at 45 ns. The equilibrium was maintained from 45 to 50 ns, after which there was a slight decline in the RMSD value for a short time, and then equilibrium was restored until the simulation run was completed. The TLR3 complex had the highest RMSD value, while the MHC-II complex had the lowest RMSD value and remained stable until the end of simulation, implying that the system is stable.

**Figure 11:**
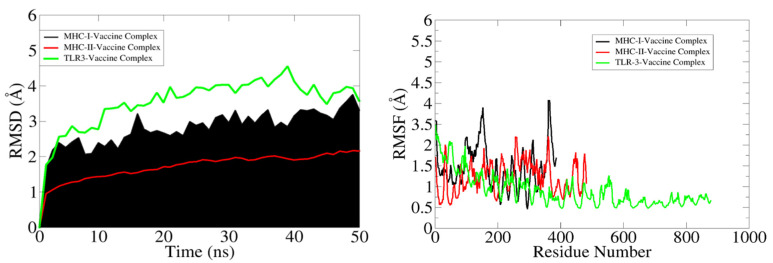
RMSD and RSMF graphs of the docked complexes (MHC-I-vaccine complex (black), MHC-II-vaccine complex (red), and TRL3-vaccine complex (green)).

The RMSF study sought to comprehend the deviations/fluctuations of all the individual residues at all the time points of the simulation study, similar to how the RMSD study focused on the complete backbone deviations. The RMSF graph was used to investigate the corresponding structural variations of complexes after knowing the nature of the flexibility of the individual residues. Gln, Asp, Gly, Cys, and Asn, which are essential residues at the binding site, show negligible changes, indicating that they are not disrupted throughout the binding process. The complete analysis of the RSMF graph revealed that the MHC-1 complex residues involved in the binding exhibit had maximum fluctuations of 4.1 nm and the residues of the MHC-II complex had maximum fluctuations of 2.2 nm, whereas the residues of the TLR3 complex had maximum fluctuations of 3.5 nm. The MHC-II complex was observed as the most stable system, but the stability of the TLR3 complex was not pronounced. The complete RMSD and RMSF study graph is shown in Figure 11.

### 3.11. Free Binding Energies

The docked complexes’ free binding energies were estimated via the MM-GB/PBSA method. The TLR3 complex’s free binding affinities were −122.17.9 kcal/mol in MMGBSA and −115.63 kcal/mol in MMPBSA. The vaccine builds for the MHC-I complex were calculated to be −125.4 kcal/mol in MMGBSA and −118.19 kcal/mol in MMPBSA, whereas the vaccine construct for the MHC-II complex was −187.94 kcal/mol in MMGBSA and −184.61 kcal/mol in MMPBSA. During the complex creation process, the net electrostatic (TLR-3-vaccine complex with 85.24 kcal/mol, MHC-I-vaccine complex −81.32 kcal/mol, and MHC-II-vaccine complex −115.17 kcal/mol) and van der Waal energies (TLR-3-vaccine complex with 67.64 kcal/mol, MHC-I-vaccine complex −69.55 kcal/mol, and MHC-II-vaccine complex −97.10 kcal/mol) are the most advantageous. All three complexes were dominated by the total energy of the gas phase (TLR-3-vaccine complex with −149.88 kcal/mol, MHC-I-vaccine complex −150.87 kcal/mol, and MHC-II-vaccine complex −212.27 kcal/mol). The polar energy, on the other hand, was unfavorable and non-polar in the complex formation (TLR-3-vaccine complex with −27.71 kcal/mol in GB and 34.25 kcal/mol in PB, MHC-I-vaccine complex 25.47 kcal/mol in GB and 32.68 kcal/mol in PB, and MHC-II-vaccine complex 24.33 kcal/mol in GB 27.66 kcal/mol in PB). Table 5 lists the various binding energy concepts.

## 4. Discussion

Despite the fact that *E. cloacae* is a typical component of animal gut flora, some strains of this bacteria are capable of causing urinary and respiratory tract infections, as well as bacteremia, septic arthritis, endocarditis, osteomyelitis, and skin infections in immunocompromised individuals. Due to the prevalence of these disorders, the commensal form of *E. cloacae* and its contributions to host immunological homeostasis have received little attention [68]. Besides cytotoxins such as enterotoxin, pore-forming toxins, hemolysins, and hemolysins, this bacterium forms biofilms in the gut, which can lead to colonization of the gut. The spread of carbapenem-resistant diseases caused by *E. cloacae* further complicates management of this pathogen. The lack of vaccine to overcome pathogen infections, as well as the lack of effective preventative measures, could result in an exponential increase in death and morbidity (https://pubmed.ncbi.nlm.nih.gov/32035109/) (accessed on 15 February 2022). As a result, a prevention approach for Enterobacter-associated illnesses is required. Vaccination is an extremely effective method for gaining protection against infectious illnesses. The development and manufacture of vaccinations based on live or attenuated viruses consumes time and significant amounts of resources. A weakened vaccination that contains a higher antigenic load, on the other hand, may overstimulate the immune system, further exacerbating the situation by causing hypersensitivity reactions [69]. Vaccines with many epitopes do not have these problems. Epitope-based immunizations have the potential to be viable choices in terms of reliability, viability, and economic feasibility. Vaccines for a wide range of viral pathogens, such as SARSCoV-2, Zika virus, Hepatitis C, CCHFV, MERS-CoV, and others, are presently under development. These vaccines serve as excellent illustrations of how a method of structural vaccinology may be used to develop a multi-epitope vaccination model.

In this investigation, in silico and immunoinformatic approaches were employed to develop an MEBPV that was effective against *Enterobacter cloacae*. Two structurally antigenic, non-allergen, and soluble proteins were selected as potential candidates for further investigation. It has previously been reported that a chimeric subunit vaccination against the *Enterobacter cloacae* reference proteome was developed, with glycoprotein serving as the immunogen. For the purpose of developing a broad-spectrum vaccination, the core proteome of 38 sequenced strains was used in this investigation. So far as *E. cloacae* strains go, this MEBPV looks more effective and immunogenic than previous MEBPV strains. A multi-epitope vaccine that is effective should be able to activate both B and T cells in the body. We were particularly interested in B cell epitopes because of their role in the establishment of memory cells and the generation of antibodies. T-cell-mediated immunity was also a source of worry during our vaccine development since plasma cells, being responsible for initiating humoral immune responses, are swiftly saturated with antigens. Furthermore, cytotoxic T cells or cell-mediated immunity may be able to provide long-term protection. Furthermore, HTL cells increase the production of interleukin-4 (IL-4), interferon-y (IFN-y), and interleukin-10 (IL-10), which help to reduce cell and tissue damage while also counteracting pro-inflammatory reactions. They also contribute to the development of IgG antibodies, and are involved in the neutralization of nosocomial illness in the peripheral nervous system. As a result, throughout the development of this vaccine, all of these considerations and evaluations were carried out.

An ideal MEBEPV would comprise B and T cell epitopes that would induce a broad immune response network, as described above. As a result, both humoral and cellular responses are generated against the vaccine. Due to an immunogenic nature, the top six epitopes were chosen for further study and investigation. It was also decided to include a GPGPG linker to join epitope–epitope sequences in order to create vaccination formulations that should be more stable [70]. Another purpose of the inserted linkers was to aid the functional preservation of epitopes (9–15 residues) after they were imported into the human body, allowing them to operate independently once they were in the body. As previously reported, the inclusion of EAAAK in the N-terminal of the fusion peptide boosts the bioactivity of the vaccine fusion protein. EAAAK was chosen as a linker since it has been shown in prior research to do so [71]. The N-terminal of the multi-epitope peptide, which had been previously designed, was attached to the B subunit of the cholera toxin as an adjuvant [72]. Its homopentameric structure and non-toxic characteristics distinguish the B subunit of the cholera toxin from other subunits. It has a strong affinity for the ganglioside monosialotetrahexosylganglioside (GM1), which can be found in a number of mammalian cells, and it is expressed by them. Macrophages, APCs, dendritic cells, and gut epithelial cells are examples of such immune cells. It also has the capability of expressing itself on its own, which results in an enhanced immune response against the antigen to which it is being linked [70].

According to earlier studies, the final MEBEPV is 208 amino acids in length, and the protein’s size is appropriate for its use as a possible vaccine design. As a result of the findings, it was concluded that the efficacy, stability, and expression of the developed MEBEPV would not be a problem during the experimental phase. As previously stated, the suggested MEBEPV was shown to be a proper immuno-dominant and was a highly antigenic non-allergen, confirming the ability of the vaccine to generate robust immune responses and minimize the risk of allergic reactions. This value demonstrates the fundamental nature of the MEBEPV, which can result in stable interactions within the range of physiological pH when calculated theoretically. The MEBEPV is thermostable and stable, as demonstrated by an estimated aliphatic and instability index; nonetheless, a negative GRAVY score suggested it to be hydrophilic, as evidenced by the presence of its strong interactions with water molecules. The peptides that have a short half-life are most difficult peptides to synthesise for therapeutic protein synthesis. According to previous reports [20], the MEBEPV developed in this study has a half-life of 30 h in the reticulocytes of mammals (in vitro), a half-life of more than 10 h in *E. coli* cells (in vivo), and a half-life of <20 h in yeast (in vivo), all of which are sufficient.

The bulk of amino acid residues showing their presence in the Ramachandran plot were seen in the favored region, and the model had a significant Z-score, implying it would be of outstanding quality, according to the data. For successful penetration in the body, the MEBPV must show a higher affinity for the immunological receptors. So, for the stimulation of the immune system and to produce vaccines and cures for dangerous viral pathogens, it is critical to have a large MHC molecule binding capability [73]. The MEBPV has a substantial MHC molecule binding potential. The initial immune responses are triggered by these encounters and are then followed by adaptive immunity due to epitopic antigens that have been exposed. The strong interactions of the MEBPV with the TLR3 and MHC II complexes, as well as other receptors, were confirmed by molecular docking and MD modelling. The analysis of MMGBSA revealed that the stable bonding requires little energy to maintain. During docking, a high number of H-bonds was observed, along with modest changes when the MD simulations were performed on the protein. As a result of these findings, the MEBV appears to have a higher affinity for the immunological receptors [6].

A multi-epitope candidate is developed by the cloning and overexpression of a multi-epitope candidate on the appropriate vector. Grasping enough knowledge of the viral results in increasing the likelihood of uncovering the critical biological features required for harmful growth would be beneficial in the creation of vaccines. As part of this investigation, in silico cloning confirms that vaccine designs were translated and expressed in the pET-28a (+expression vector). The MEBPV design comprised T cell and B cell epitopes, suggesting that it is crucial to elicit immunological responses in a host by activating various immune cells by complex signaling [74]. The only disadvantage of the current study is that it does not include any experimental validations. It is dependent on experimental procedures to generate the first raw data that are used for further analysis in bioinformatics approaches. The effectiveness of the computational methodologies, as well as the quality of the data utilized in the immunoinformatic forecasts, can be used to determine the efficiency of these forecasts, and experimental studies are further necessary to determine the full potential of the MEBPV intended to combat *E. cloacae*.

## 5. Conclusions

The bacteria belonging to the family Enterobacteriaceae have arisen as problematic to public health, and efforts are being made to tackle these infections. With the help of a pan-genome, subtractive proteomics, and immunoinformatic techniques, we developed a safe and effective vaccination against 39 sequenced strains of the pathogenic species *E. cloacae* that were resistant to antibiotics. The vaccine design has been demonstrated to bind with high affinity to the immune receptors MHC-I, MHC-II, and TLR-3, resulting in a rapid response and the establishment of effective adaptive immunity against the pathogen. The results of the dynamics modelling investigations revealed that the intermolecular contacts were quite stable, demonstrating that the expected binding mechanism of the construct was correct. Finally, free energies calculations are used to supplement the findings of the docking and simulation experiments in order to decode the high stability of the complex. However, while our in silico studies indicate that the vaccine ensemble is immunogenic, it is unclear if it will confer protection against *E. cloacae* infection to any significant level. Overall, this was an excellent example of in silico reverse vaccinology in the context of screening antigens with the largest range of bactericidal action. Furthermore, it is highly recommended that in vivo research be carried out to validate the in silico data, such as the effect of vaccine and the vaccination on bacterial load, serum cytokine levels, serum IgG antibody titers, and histological examinations of the process in Balb/c mice. To give an example, in silico approaches were used to predict a potential vaccine target (FilF) for the *Acinetobacter baumannii* superbug (*A. baumannii*). FilF vaccination of the mouse pneumonia model resulted in a high antibody titer (>64,000) following the immunization. In addition, a mouse survival rate of around 50% was observed when they were exposed to a fatal dosage of *A. baumannii* (108 CFU). The bacterial burden was discovered to be lowered by two and four log cycles, respectively. The amount of pro-inflammatory cytokines was found to be much lower, which was corroborated by a reduction in neutrophil infiltration and tissue damage in the lung. The designed vaccine construct consisted of six probable antigenic epitopes: KNDRTDVKT, KADGEGDKA, DGDAGFANK, QGKNDNRNE, KDLYARNGY, and LLDEEDGAI. To summarize, these results show that an immune-protective protein predicted by in silico proteomic screening has immuno-protective activity in the body. It is now possible to apply experimental research on the designed vaccine model to validate the predictions.

## Figures and Tables

**Table 1:** Properties of the proteins used for the prediction of the immunodominant epitopes.

Accession No	Protein	T.Helices	MW	Instability Index	Antigenicity 0.7	Adhesion 0.6	Allergenisity	Solubility	Blastp (Human)	Blastp (Lactobacillus)
BBS36577.1	Phosphoporin PhoE	1	40.285.2	10.49	0.8008	0.896	Non-allergen	Soluble	No similarity Found	No similarity Found
WP_038419991.1	Porin	0	39.92468	17.82	0.7422	0.633	Non-allergen	Soluble	No similarity Found	No similarity Found

**Table 2:** Selection of the potent epitopes for the vaccine construct and the parameters for evaluation of the epitopes.

Proteins	B Cell Epitopes	MHC I	P.Rank	MHC II	P.Rank	MHC Pred	Score	Antigenisity	Allergenicity
**Phosphoporin PhoE**	YQGKNDRTDV KTANGDGVGY	KNDRTDVKTANG	28	KNDRTDVKTA	7.8	KNDRTDVKT	13.06	1.7238	non-allergen
	SYSNANRTLKQK ADGEGDKAEA	KADGEGDKAEA	37	KADGEGDKA	3.4	KADGEGDKA	42.95	3.0005	non-allergen
	AETRNTTRTGT DGDAGFANKT	TGTDGDAGFANKT	14	DGDAGFANKT	13	DGDAGFANK	65.31	0.919	non-allergen
**Porin**	YQGKNDNRNE FKANGDG	YQGKNDNRNEF	47	YQGKNDNRNE	24	QGKNDNRNE	59.29	2.3164	non-allergen
	QGKDLYARNG YKGVDAD	GKDLYARNGYKGVD	16	KDLYARNGYK	0.07	KDLYARNGY	36.56	1.203	non-allergen
	NLLDEEDGAIT GNATD	NLLDEEDGAITGN	33	LLDEEDGAI	1.16	LLDEEDGAI	36.81	0.8689	non-allergen

**Table 3:** Structural features of the five models generated after refining the multi-epitope vaccine structure.

Model	GDT-HA	RMSD	Molprobity	Clash Score	Poor Rotamers	Rama Favored
Initial	1	0	2.945	46.7	2.5	91.7
MODEL 1	0.9339	0.444	1.8	11.1	1.3	97.1
MODEL 2	0.9471	0.418	1.654	9.2	1.3	97.6
MODEL 3	0.9315	0.476	1.74	9.5	1.3	97.1
MODEL 4	0.9339	0.46	1.627	8.6	1.3	97.6
MODEL 5	0.9435	0.435	1.604	9.8	0.6	97.6

**Table 4:** Docking statistics of the designed vaccine construct to MHCI, MHCII, and TLR3 receptors, with energy given in kJ/mol.

Docking Statistics	Global Energy	Attractive VdW	Repulsive VdW	Atomic Contact Energy	Hydrogen Bond Energy
MHC I	−18.64	−32.11	11.75	3.46	−2.04
MHC II	−48.25	−40.61	25.59	2.59	−1.70
TLR3	−5.20	−28.10	13.08	17.38	−7.28

**Table 5:** MM-GBSA-based energy parameters of the complexes (TLR3-vaccine, MHC I-vaccine, and MHC II-vaccine), with the net delta energy given in kcal/mol.

Energy Parameter	TLR3-Vaccine Complex	MHC I-Vaccine Complex	MHC II-Vaccine Complex
MM-GBSA			
VDWAALS	−67.64	−69.55	−97.10
EEL	−85.24	−81.32	−115.17
Delta G gas	−149.88	−150.87	−212.27
Delta G solv	27.71	25.47	24.33
Delta Total	−122.17	−125.4	−187.94
MM-PBSA			
VDWAALS	−67.64	−69.55	−97.10
EEL	−85.24	−81.32	−115.17
Delta G gas	−149.88	−150.87	−212.27
Delta G solv	34.25	32.68	27.66
Delta Total	−115.63	−118.19	−184.61

## Data Availability

The data presented in this study are available within the article.
